# SEOM-GETNE-TTCC Clinical guideline thyroid cancer (2023)

**DOI:** 10.1007/s12094-024-03736-6

**Published:** 2024-09-26

**Authors:** Teresa Alonso-Gordoa, Paula Jimenez-Fonseca, Javier Martinez-Trufero, Miguel Navarro, Ignacio Porras, Jordi Rubió-Casadevall, Marta Arregui Valles, Neus Basté, Jorge Hernando, Lara Iglesias Docampo

**Affiliations:** 1 Medical Oncology Department, Hospital Universitario Ramón y Cajal, Madrid, Spain; 2 Medical Oncology Department, Hospital Universitario Central de Asturias, ISPA, Oviedo, Spain; 3 Medical Oncology Department, Hospital Universitario Miguel Servet, Zaragoza, Spain; 4 Medical Oncology Department, Complejo Asistencial Universitario de Salamanca. IBSAL, Salamanca, Spain; 5 Medical Oncology Department, Hospital Universitario Reina Sofía, Instituto Maimónides de Investigación Biomédica de Córdoba (IMIBIC), Córdoba, Spain; 6 Medical Oncology Department, Catalan Institute of Oncology of Girona, Hospital Josep Trueta. ONCOGIRPRO Group, Girona Biomedical Research Institute (IDIBGI), Girona, Spain; 7 Medical Oncology Department, Hospital General Universitario Gregorio Marañón, Madrid, Spain; 8 Medical Oncology Department, Hospital Clinic Barcelona, IDIBAPS, Barcelona, Spain; 9 Medical Oncology Department, Gastrointestinal and Endocrine Tumor Unit, Hospital Universitario Vall D’Hebron, VHIO, Barcelona, Spain; 10 Medical Oncology Department, Hospital Universitario 12 de Octubre, Madrid, Spain

**Keywords:** Thyroid carcinoma; Molecular alterations; Multidisciplinary; Systemic treatment

## Abstract

Thyroid cancer (TC) represents 3% of global cancer incidence. Recent changes have optimized treatment decisions based on risk assessment, molecular profiling, and imaging assessment, leading the development of targeted agents that have modified the natural history of this disease. This increasing complexity on treatment options requires careful assessment at the different stages of the disease to provide the most suitable approach from diagnosis to long-term follow-up. This guideline aims to offer a comprehensive and practical overview on the current status and last updates of TC management.

## Incidence and epidemiology

Thyroid cancer (TC) accounts for 3% of global cancer incidence and is the most common endocrine malignancy. In 2022, 821.173 new cases were estimated worldwide, with differences by geographical area, age, and sex, although it only constitutes 0.5% of mortality, with an estimated number of 47.485 cases [1].

Focusing on estimated data from Spain, European standardized incidence rates of TC in 2020 are 16.2 cases/100.000 inhabitants in women and 4.7 in men. Mortality rates are similar in both sexes, 0.7 cases/100.000 inhabitants in women and 0.6 in men [2]. Estimated number of new thyroid cancer cases in Spain for 2023 was 6084 (*Red Española de Registros de Cáncer*) [3].

Using data from European cancer registries, in women, papillary thyroid carcinoma (PTC) constitutes 73% of all cases, follicular thyroid carcinoma (FTC) 14%, medullary thyroid carcinoma (MTC) 3%, anaplastic/undifferentiated carcinoma 3%, other histologies 2% and unspecified histology 4%. In men, this distribution accounts 64%, 16%, 7%, 4%, 3% and 6%, respectively [4].

A worldwide increase in the incidence of PTC in the last thirty years has been described. Implementation of cancer screening, better diagnostic accuracy and availability of neck ultrasound (US) or other imaging studies could be the reasons that explains this phenomenon. This leads to a growing diagnosis of clinically asymptomatic low-risk PTCs, which probably does not influence in patient’s survival. Differences in incidence between European countries are almost exclusively limited to PTC, which suggests that the patterns of overdiagnosis of this histology are probable culprits for this fact [4].

The only well-established risk factor for follicular cell-derived TC is ionizing radiation, particularly exposure during childhood. Other factors (obesity, smoking, hormonal exposures, familial history, and certain environmental pollutants) may also contribute [5]. For MTC, the risk associated to germline RET mutations in the context of hereditary syndromes, such as MEN2 (Multiple Endocrine Neoplasia 2), is between 80 and 100%.

While most TC patients have an excellent prognosis, with a 5-year relative survival (5-y RS) rate of 98.2% for all stages; 99.9% in localized, and 98.2% for regional stages according to SEER data from the 2009–2015 cohort, the prognosis for distant disease drops to 56.2% [6]. According to histological subtype, TC patients diagnosed in Europe between 2000 and 2007 showed a 5-y RS rate for all stages of 96% in PTC, 91% in FTC, 88% in MTC and 14% in anaplastic carcinoma (including patients with poorly differentiated TC, PDTC) in women and 92%, 87%, 75% and 11%, respectively in men [7]. Recent studies have reported data on PDTC prognosis, as a distinct entity from ATC, with a 5-y OS of 85% and 5-y DSS of 66% [8].

## Methodology

This guideline is based on a systematic review of relevant published studies and with the consensus of ten treatment expert oncologists from GETNE (Grupo Español de Tumores Neuroendocrinos y Endocrinos), TTCC (Grupo Español de Tumores de Cabeza y Cuello) and SEOM (Spanish Society of Medical Oncology), and an external review panel of two experts designated by SEOM. The Infectious Diseases Society of America-US Public Health Service Grading System for Ranking Recommendations in Clinical Guidelines has been used to assign levels of evidence and grades of recommendation (Table 1).

**Table 1:** Quality of evidence and strength of recommendation score

Quality of evidence	
I	Evidence from at least one large randomized, controlled trial of good methodological quality (low potential for bias) or meta-analyses of well-conducted randomized trials without heterogeneity
II	Small randomized trials or large randomized trials with a suspicion of bias (lower methodological quality) or meta-analyses of such trials or of trials with demonstrated heterogeneity
III	Prospective cohort studies
IV	Retrospective cohort studies or case–control studies
V	Studies without control group, case reports, expert opinions
Strength of recommendation	
A	Strong evidence for efficacy with a substantial clinical benefit, strongly recommended
B	Strong or moderate evidence for efficacy but with a limited clinical benefit, generally recommended
C	Insufficient evidence for efficacy or benefit does not outweigh the risk or the disadvantages (adverse events, costs, etc.), optional
D	Moderate evidence against efficacy or for adverse outcome, generally not recommended
E	Strong evidence against efficacy or for adverse outcome, never recommended

## Diagnosis, pathology, and molecular biology

The standard diagnostic process for a patient with a suspicious thyroid nodule involves blood tests to check thyroid function, US scans, and a fine-needle aspiration cytology (FNAC) test [9]. A nodule might be suspected to be cancerous by US when it is found to be solid, with low echo patterns, a height larger than its width, increased blood flow, tiny calcium deposits, and/or with uneven edges [9, 10]. The American Thyroid Association (ATA) classifies US results into five risk levels, from benign to highly suspicious. The highest risk level calls for an FNAC test [10]. FNAC is also recommended for thyroid nodules > 1 cm or smaller nodules associated with clinical risk factors, such as previous radiation, family history, or nearby swollen lymph nodes. For US low-risk nodules, FNAC is suggested if they measure > 2 cm.

According to the Bethesda classification based on FNAC findings, there are six scenarios, ranging from non-diagnostic/unsatisfactory (Bethesda I) to a confirmed cancer diagnosis (Bethesda VI) [11]. TC can often be diagnosed with cytology, but follicular types differ from adenomas by vascular or capsular invasion, which can't be assessed with cytology.

After confirming TC, computed tomography (CT) complements the neck US only in patients with clinical suspicion for invasive primary tumor, or clinically apparent multiple or bulky lymph node involvement [10]. Serum markers include thyroglobulin (Tg) and anti-thyroglobulin antibodies (TgAb) for follicular cell-derived thyroid carcinoma, and calcitonin and CEA for MTC.

**Fig. 1:**
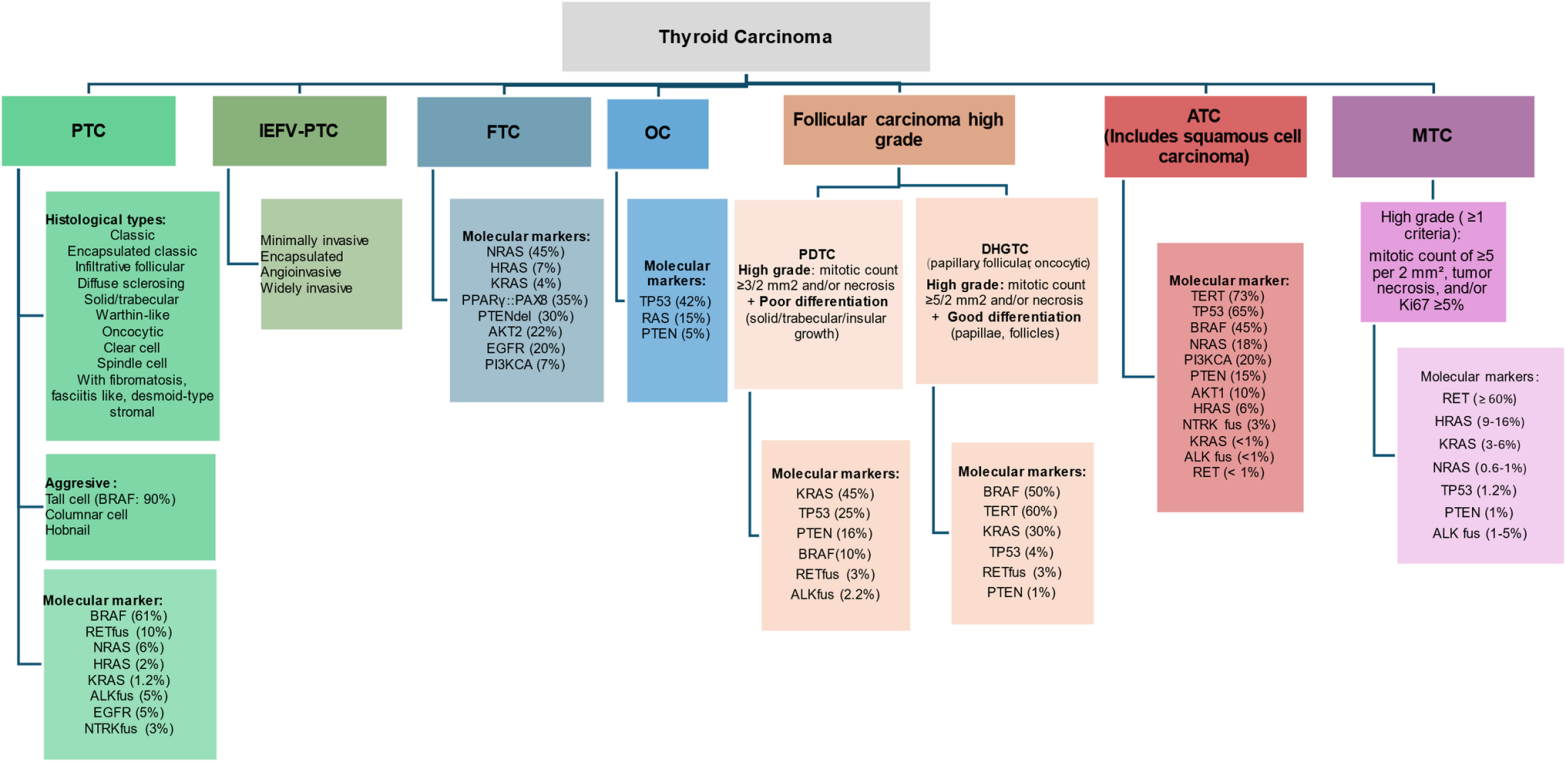
Histological types of TC and principal molecular markers. *FTC* follicular thyroid carcinoma; *PTC* papillary thyroid carcinoma; *IEFV-PTC* invasive encapsulated follicular variant of papillary thyroid carcinoma; *OC* oncocytic carcinoma; *PDTC* poorly differentiated thyroid carcinoma; *DHGTC* differentiated high-grade thyroid carcinoma; *ATC* anaplastic thyroid carcinoma; *del* deletion; *fus* fusion; *m* mutation. (if not specified, molecular alterations are mutations)

In the fifth edition of the WHO Classification, each tumor is classified based on its cell of origin, pathological or molecular features, and biological behavior (Fig. 1) [12]. Among the malignant neoplasms derived from follicular cells are PTC, FTC, anaplastic (ATC), oncocytic (OC, previously known as Hürthle cell carcinoma), invasive encapsulated follicular variant of papillary thyroid carcinoma (IEFV-PTC), and the high-grade carcinoma [12]. The squamous thyroid carcinoma, which was listed separately in the previous edition, is now considered a histological pattern of ATC with a higher BRAF mutation rate and PAX8 positivity [12].

Encapsulated follicular carcinomas include FTC, OC, and IEFV-PTC. For prognostic purposes, these tumors are categorized into minimally invasive (only capsular invasion), encapsulated angioinvasive (limited vascular invasion (< 4 foci) or extensive invasion (≥ 4 foci)), and widely invasive (the tumor capsule is entirely obliterated or partially intact and/or with evident invasion across the gland) [12].

High-grade follicular cell-derived carcinoma has two histological subtypes: traditional poorly differentiated (PDTC) and differentiated high-grade (DHGTC). These are determined based on mitotic count and the presence of necrosis, without considering the Ki67 index. PDTC has a mitotic count of ≥ 3 per 2 mm^2^, while DHGTC has ≥ 5 mitoses per 2 mm^2^ [12].

MTC is divided into low- and high-grade subtypes. A high-grade carcinoma should exhibit at least one of the following features: mitotic count of ≥ 5 per 2 mm^2^, tumor necrosis, and/or Ki67 ≥ 5% and has been associated with worse clinical outcomes increasing the risk of relapse and mortality [12].

PTC includes the histological subtypes shown in Fig. 1. Aggressive PTC subtypes comprise tall cell (with ≥ 30% of cells being at least three times taller than their width, dense eosinophilic cytoplasm, and distinct cell membranes), columnar cell, and hobnail PTC (with mixed papillary and micropapillary structure, discohesive tumor cells, and nuclear atypia) [12]. Aggressive PTCs typically present in older age, are often associated with angiovascular invasion, advanced pathological stage, and display increased mitotic activity.

The most common molecular alterations for each histological type of TC are shown in Fig. 1 [13, 14]. All patients with TC should have access to somatic molecular profiling, preferably based on Next-Generation Sequencing (NGS) due to the availability of effective targeted drugs [15]. The optimal timing for molecular testing will depend on patient evolution and test availability. Ideally, it should be performed before the systemic treatment in MTC and ATC and, at least, during the first-line MKI treatment in DTC, before disease progression. In selected patients, to optimizing molecular analysis procedures (long time from primary diagnosis) and results, it can be considered the necessity of performing a new biopsy of a metastatic lesion.

To rule out the possibility of a MEN2 syndrome in the family, all patients with MTC should be tested for germline RET mutations and offered genetic counseling [16].

Recommendations

- If a thyroid nodule is found during a physical exam, a neck US and thyroid function tests should be performed (Level of evidence: I, Recommendation grade: A).
- Any clinically suspicious thyroid nodule should also receive a FNAC (Level of evidence: II, Recommendation grade: A).
- For TC, neck node staging is done with US, and distant staging with CT in selected cases (Level of evidence: II, Recommendation grade: A).
- All patients with MTC should be tested for germline RET mutations and offered genetic counseling. (Level of evidence: II, Recommendation grade: A).
- Patients with TC needing systemic treatment should have access to somatic molecular profiling, preferably based on NGS due to the availability of effective targeted drugs. (Level of evidence: III, Recommendation grade: B).

## Staging and risk assessment

Two staging systems are the most used in TC: the American Joint Committee on Cancer (AJCC), tumor, node, and metastases (TNM) staging systems to estimate disease-specific mortality and the ATA system that estimates the risk of persistent or recurrent TC (Table 2) [10, 17].

**Table 2:** ATA risk stratification system to estimate risk of persistent/recurrent disease

Low risk
Papillary thyroid cancer with all of the following present:
• No local or distant metastases
• All macroscopic tumor has been resected
• No invasion of locoregional tissues
• Tumor does not have aggressive histology (aggressive histologies include tall cell, insular, columnar cell carcinoma, oncocytic carcinoma, follicular thyroid cancer, hobnail variant)
• No vascular invasion
• No 131I uptake outside the thyroid bed on the post-treatment scan, if done
• Clinical N0 or ≤ 5 pathologic N1 micrometastases (< 0.2 cm in largest dimension) *
Intrathyroidal, encapsulated follicular variant of papillary thyroid cancer* Intrathyroidal, well-differentiated follicular thyroid cancer with capsular invasion and no or minimal (< 4 foci) vascular invasion* Intrathyroidal, papillary microcarcinoma, unifocal or multifocal, including BRAF V600E-mutated (if known) *

Intermediate risk
Any of the following present:
• Microscopic invasion into the perithyroidal soft tissues
• Cervical lymph node metastases or 131I avid metastatic foci in the neck on the post-treatment scan done after thyroid remnant ablation
• Tumor with aggressive histology or vascular invasion (aggressive histologies include tall cell, insular, columnar cell carcinoma, oncocytic cell carcinoma, follicular thyroid cancer, hobnail variant)
• Clinical N1 or > 5 pathologic N1 with all involved lymph nodes < 3 cm in largest dimension*
• Multifocal papillary thyroid microcarcinoma with extrathyroidal extension and BRAF V600E-mutated (if known) *

High risk
Any of the following present:
• Macroscopic tumor invasion
• Incomplete tumor resection with gross residual disease
• Distant metastases
• Postoperative serum thyroglobulin suggestive of distant metastases
• Pathologic N1 with any metastatic lymph node ≥ 3 cm in largest dimension*
• Follicular thyroid cancer with extensive vascular invasion (> 4 foci of vascular invasion) *

Adapted from reference 18. *Proposed modifications, not present in the original 2009 initial risk stratification system.

The TNM 8th edition incorporated relevant changes: it increased the cut-off age at diagnosis to 55 years, downstaged extrathyroidal extension that was not macroscopically evident (pT3b), included mediastinal lymph nodes as N1a, considered N1b for metastatic lymph nodes at cervical levels II–V, and no longer upstaged N1 disease in older patients to stage III or IV. Therefore, in patients older than 55 years, N1 disease is classified as stage II disease [17].

ATA guidelines were revised in 2015 and the recurrence risk classification system was modified by adding additional variables available after treatment of the primary tumor including the size and number of lymph node metastases, vascular invasion, specific histologies, and molecular profiling [10, 18]. According to these characteristics, TC is classified as either low, intermediate, or high risk for recurrence.

Additionally, the dynamic risk stratification approach allows a more accurate risk assessment than the static estimate from the TNM or ATA systems and several studies have demonstrated its clinical usefulness [18]. In this system, patients are classified according to response to initial therapy and biological behavior of the disease, as having one of the following clinical outcomes [19]:

- Excellent response: no clinical, biochemical, or structural evidence of disease.
- Biochemical incomplete response: abnormally elevated serum Tg or TgAb levels in the absence of localizable disease.
- Structural incomplete response: persistent or newly identified locoregional or distant metastases with or without abnormal Tg or TgAb.
- Indeterminate response: nonspecific biochemical or structural findings that cannot be confidently classified as either benign or malignant.

## Management of local and locoregional disease

### Differentiated thyroid carcinoma

#### Surgical treatment

**Fig. 2:**
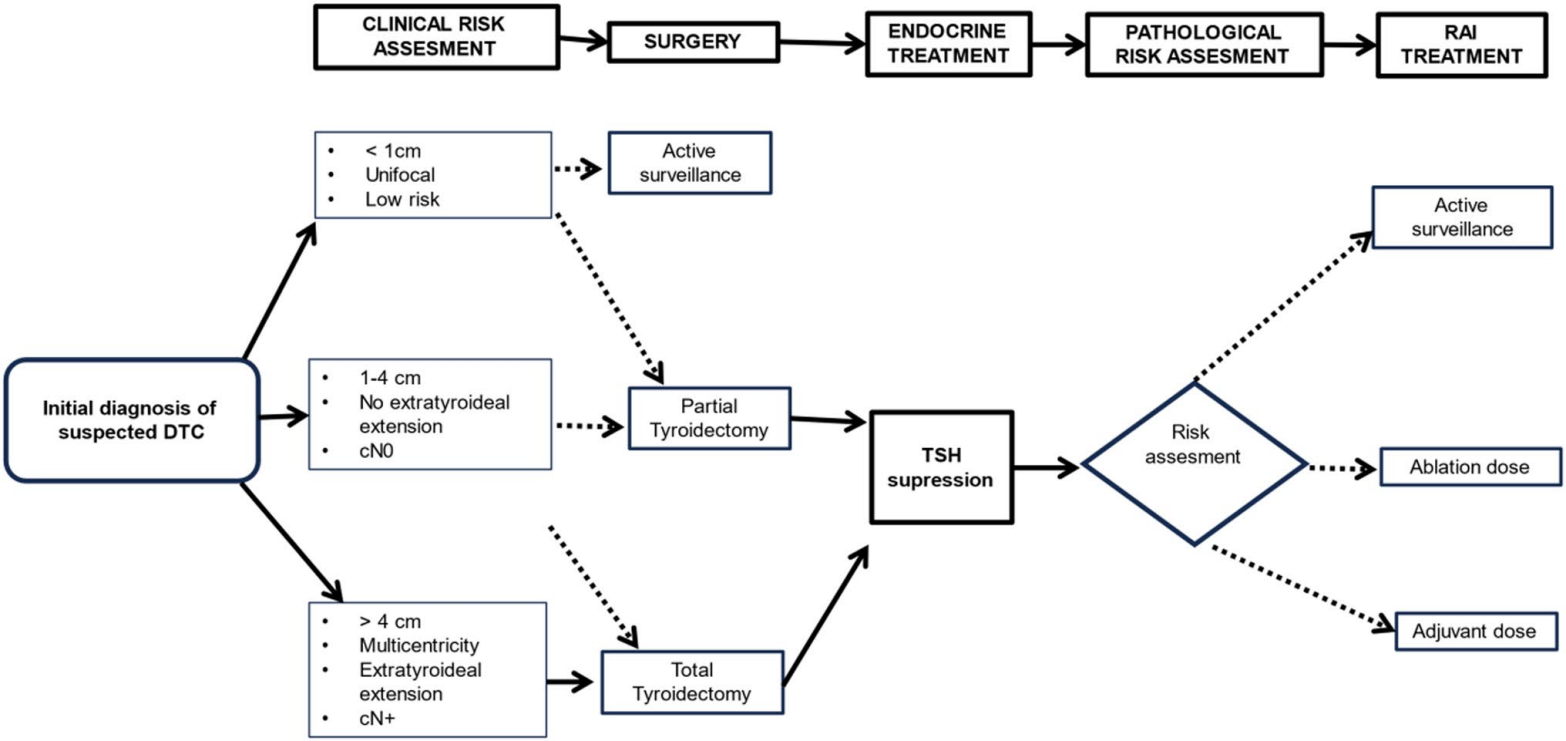
Basic therapeutic itinerary of patients with localized *DTC*. *DTC* Differentiated Thyroid Carcinoma, *cN0*node-negative, *cN* + node-positive, *TSH* thyroid-stimulating hormone, *RAI* radioactive iodine

The extent of surgical resection in DTC remains under debate. Total thyroidectomy is widely recommended in patients with nodules greater than 4 cm, since it is associated with an improvement in disease-free survival, overcomes the possibility of multicentricity in PTC (30–40% of patients), allows post-operative radioactive iodine 131I (RAI), and serum levels of Tg are more useful for monitoring in long-term follow-up (Fig. 2) [10].

For patients with TC nodules between 1 and 4 cm, with no extrathyroidal extension, and no clinical evidence of any lymph node metastases (cN0), the initial surgical approach can be either bilateral (near-total or total thyroidectomy) or unilateral procedure (lobectomy), based on data from most recent studies (Fig. 2) [20].

In patients with small (< 1 cm), low-risk, unifocal, intrathyroidal PTC, partial thyroidectomy can be enough since no differences in survival have been reported when compared with total thyroidectomy (Fig. 2) [20]. In recent years, active surveillance (AS) has been proposed as an alternative approach to surgery. It has been suggested as a reasonable option, particularly in patients older than 60 years with previously mentioned criteria, surgical risk, and when a strict compliance of follow-up every 6–12 months can be guaranteed [10, 21]. Data from a recent meta-analysis reported that only 12% of patients under AS finally underwent surgery, and only 2% developed lymph node metastases [21]. Alternatively, minimally invasive ablation techniques may be considered in selected patients who prefer proactive measures over AS [22]. Neither partial thyroidectomy nor AS should be considered in case of contralateral thyroid nodules, aggressive cytological characteristics, previous head or neck irradiation or familial thyroid cancer [20].

Therapeutic lymphadenectomy should be performed in the central and/or lateral compartments when there is clinical or radiographic evidence of lymph node involvement [10]. The role of prophylactic central compartment neck dissection (pCCND) in patients with clinically unaffected lymph nodes in the central compartment (cN0) is still under debate. Therefore, results of two large meta-analysis have shown that patients with pCCND have lower rates of locoregional recurrence although suffering higher morbidity [23, 24]. This procedure is currently accepted in patients cN0 who have advanced primary tumors, such as T3–T4, or clinically involved lateral neck nodes, or if the information will be used to plan further steps in therapy [10, 25]. The number of nodes that should be resected in a pCCND is not well-established, but it seems that at least 6 nodes should be examined to reach a false-negative likelihood rate lower than 10% [26]. For unresectable or high-risk R1/R2 resections, neoadjuvant therapy with targeted therapy is under research [27].

#### Postoperative adjuvant therapy

Risk for local and distant recurrence depends on several clinical–pathological factors obtained perioperatively. There are primary basic factors, such as basic histology, age at diagnosis, primary tumor size, lymph node metastasis, extrathyroidal extension, invasion of structures, residual post-operative disease, distant metastasis, post-operative Tg levels, RAI uptake outside thyroid bed. Besides that, there are other secondary factors (pathological and molecular) that may be considered within the context of experienced multidisciplinary team board committees (MTBC) [28]. Based on these factors, there are mainly two different treatments aimed to eliminate residual remnant thyroid tissue and neoplastic cells, thereby reducing the risk of recurrence and mortality (Fig. 2):

- Thyroid-stimulating hormone (TSH) suppressive therapy (serum TSH < 0.1 mU/l) with levothyroxine (LT4): mandatory in patients with evidence of persistent disease to regulate the postsurgical hypothyroidism and to inhibit the TSH-dependent growth of residual cancer cells [29].
- Postoperative 131I RAI: this treatment should be administered after TSH stimulation by withdrawing LT4 for 4–5 weeks or by administering recombinant human TSH (rhTSH) to increase RAI uptake. It can be given with two different dose schedules:
  o Ablative low RAI dose (30 mCi, 1.1 GBq): this approach eliminates post-operative residue of benign thyroid tissue and facilitates follow-up studies. It is considered in selected low-risk patients according to an MTBC assessment.
  o Adjuvant high RAI dose (≥ 100 mCi, 3.7 GBq): considered mainly in high-risk patients (Level of evidence III; Grade of recommendation A).
  o Adjuvant RAI dose (30–100 mCi, 1.11–3.7 GBq) decision should be considered on a MTBC assessment in intermediate and selected low-risk patients due to controversial results from retrospective studies [30]. In intermediate-risk patients, retrospective studies have identified major benefit from adjuvant RAI dose in patients with advanced age (≥ 55 years old), aggressive histology and extended lymph node metastases. In low-risk patients, routine RAI is not administered in patients with pT1a, multifocal (< 1 cm) or intrathyroid tumors. The phase III randomized ESTIMABL2 trial showed that a follow-up strategy in those patients was non-inferior to post-operative radioiodine (1.1 GBq). However, this conclusion was based on a follow-up of 3 years, quite short for this population. Patients included in the trial had selected multifocal pT1a or a pT1b tumors and/or N0/Nx without extrathyroidal extension or aggressive histologies. Current trials addressing this scenario are undergoing (IoN and CLERAD-PROBE) and results may clarify RAI indication in this population [31].

Recommendations

- Total thyroidectomy is recommended in patients with nodules greater than 4 cm in DTC. (Level of evidence: IV, grade of recommendation: A).
- For nodules between 1 and 4 cm, without extrathyroidal extension and cN0, near-total or total thyroidectomy, or lobectomy can be considered (Level of evidence: IV, grade of recommendation: B).
- In patients with small, low-risk DTC, partial thyroidectomy may be considered, however, AS is also an option (Level of evidence: II, grade of recommendation: B).
- Therapeutic lymphadenectomy should be performed when lymph node involvement (Level of evidence: II, grade of recommendation: A).
- There are two treatments aimed at reducing the risk of recurrence and mortality after surgery:
  ➤ Thyroid-stimulating hormone (TSH) suppressive therapy: (Level of evidence: IV, grade of recommendation: B).
  ➤ Postoperative radioactive iodine 131I (RAI):
    o High-risk patients: Adjuvant RAI dose is recommended (Level of evidence IV, grade of recommendation A).
    o Intermediate- and low-risk patients: Postoperative adjuvant RAI dose should be individualized based on a MTBC assessment (Level of evidence IV, grade of recommendation B).
    o In selected low-risk patients, ablative low RAI dose can be considered (Level of evidence IV, grade of recommendation C).

### Anaplastic thyroid carcinoma

ATC is rarely amenable to complete resection. In those few cases of localized resectable ATC, total thyroidectomy with bilateral central neck dissection should be performed. Incomplete palliative resection (R2) or debulking is not recommended [32]. Tracheostomy may be needed to alleviate symptoms and provide time to carry out molecular studies and be able to start active treatments.

For patients with tumors that are difficult to resect or require extensive surgery (stage IVb) and who have the BRAF V600E mutation, neoadjuvant dabrafenib plus trametinib can improve the chances of achieving a complete tumor resection with a reduced extent of surgery and lower surgical morbidity [33]. Also, for patients without BRAF V600E mutation, initial results are showing activity in this setting for the combination of MKI ± PD-1 inhibitors and a phase II trial is currently ongoing (NCT04321954) [34].

Post-surgical Radiotherapy (RT) is required to obtain better outcomes in terms of survival and local disease control after complete (R0) or near complete (microscopic residual tumor, R1) resection [35]. Intensity-modulated RT (IMRT) is the recommended approach because of the improved dosed distribution and the ability to reduce toxicity. A multimodal approach with concomitant chemotherapy (usually weekly doxorubicin, paclitaxel, or platinum agents), may improve survival outcomes in selected patients with adequate performance status [32].

For patients with unresectable tumors and for whom targeted therapy is not feasible, concomitant RT and chemotherapy with the previously mentioned regimens may be considered in fit patients. Depending on the response achieved, a local approach may be reassessed [32].

Recommendations

Timely discussion of ATC patients by a MTBC and local and/or systemic treatment initiation is strongly recommended.

- Total thyroidectomy with lateral and/or central node dissection should be performed in resectable non-metastatic ATC (Level of evidence: IV, Grade of recommendation: A).
- Neoadjuvant dabrafenib plus trametinib in patients harboring BRAF V600E mutation improve the chances of future complete tumor resection with reduced extent of surgery and surgical morbidity (Level of evidence: V; Grade of recommendation: A).
- Adjuvant RT (IMRT) with or without concomitant chemotherapy should be offered to selected patients with adequate performance status (Level of evidence: IV; Grade of recommendation B).
- Concomitant RT and chemotherapy should be offered in fit patients with unresectable stage IVB or IVC tumors without a targetable molecular alteration (Level of evidence: IV; Grade of recommendation B).

### Medullary thyroid carcinoma

Total thyroidectomy and bilateral central neck dissection (level VI), with additional lateral neck compartments dissection if there is evidence of involvement (levels II–VI) is the recommended approach [36].

For patients with unilateral and < 1 cm tumors, prophylactic bilateral central neck dissection is not required if preoperative calcitonin level < 20 pg/mL, as metastatic lymph nodes are rare in this scenario [37].

For patients with no clinical evidence of lateral lymph node metastases on the preoperative neck US, the role of prophylactic lateral compartment dissection is controversial and there is a trend to de-escalating surgical extent. Furthermore, local recurrence is not related to distant recurrence and does not adversely impact overall survival (OS) [38]. Residual lymph node metastases after thyroidectomy, based on calcitonin levels, imaging studies or inadequate surgery could benefit from reoperation with minimal morbidity [39].

According to calcitonin levels, and based on expert recommendations, contralateral neck dissection may be considered if calcitonin levels are greater than 200 pg/mL and if preoperative imaging in the ipsilateral neck compartment is positive [37, 40].

Based on retrospective studies, there is a benefit in local recurrence for RT in patients with extensive local disease or with metastatic disease where a less aggressive local approach is preferred in combination with other strategies [36].

Recommendations

- Total thyroidectomy, bilateral central neck dissection and involved lateral neck compartments are recommended for MTC with neck confined ≥ 1 cm or when bilateral thyroid disease is present, based on neck imaging (Level of evidence: III; Grade of recommendation: B).
- For calcitonin levels above 200 pg/mL bilateral modified radical neck dissection may be indicated when preoperative imaging is positive in the ipsilateral neck compartment. (Level of evidence: IV; Grade of recommendation: C).

## Management of advanced and metastatic disease

### Differentiated thyroid carcinoma

#### Initial treatment

Despite metastatic synchronous spread, locoregional approach with primary tumor resection is important to optimize RAI activity and improve local control [10].

First-line treatment in patients with RAI-avid tumors is RAI [41]. However, one-third of patients will present non-avid lesions and will be classified as RAI-refractory (RAI-R).

RAI-R status criteria include an initial absence of RAI uptake, a progressive decline in RAI uptake following RAI, heterogeneity in uptake, and progression after subsequent RAI. Other special situations could be considered: Uptake in 18FDG-PET, unresectable primary tumor, and aggressive histologies [42].

Not all patients require immediate systemic treatment upon RAI-R status. An individualized treatment strategy should consider disease evolution, potential toxicities, comorbidities, and patients´ preferences. Certain variables like Tg doubling time, radiology progression by RECIST, or lung metastasis volume doubling time can help in this clinical decision [43]. Systemic treatment could be delayed with locoregional treatments in cases of oligometastatic spread. Radiotherapy is an option in symptomatic bone metastasis and bisphosphonates or denosumab can be considered [44].

#### Multikinase inhibitors

**Fig. 3:**
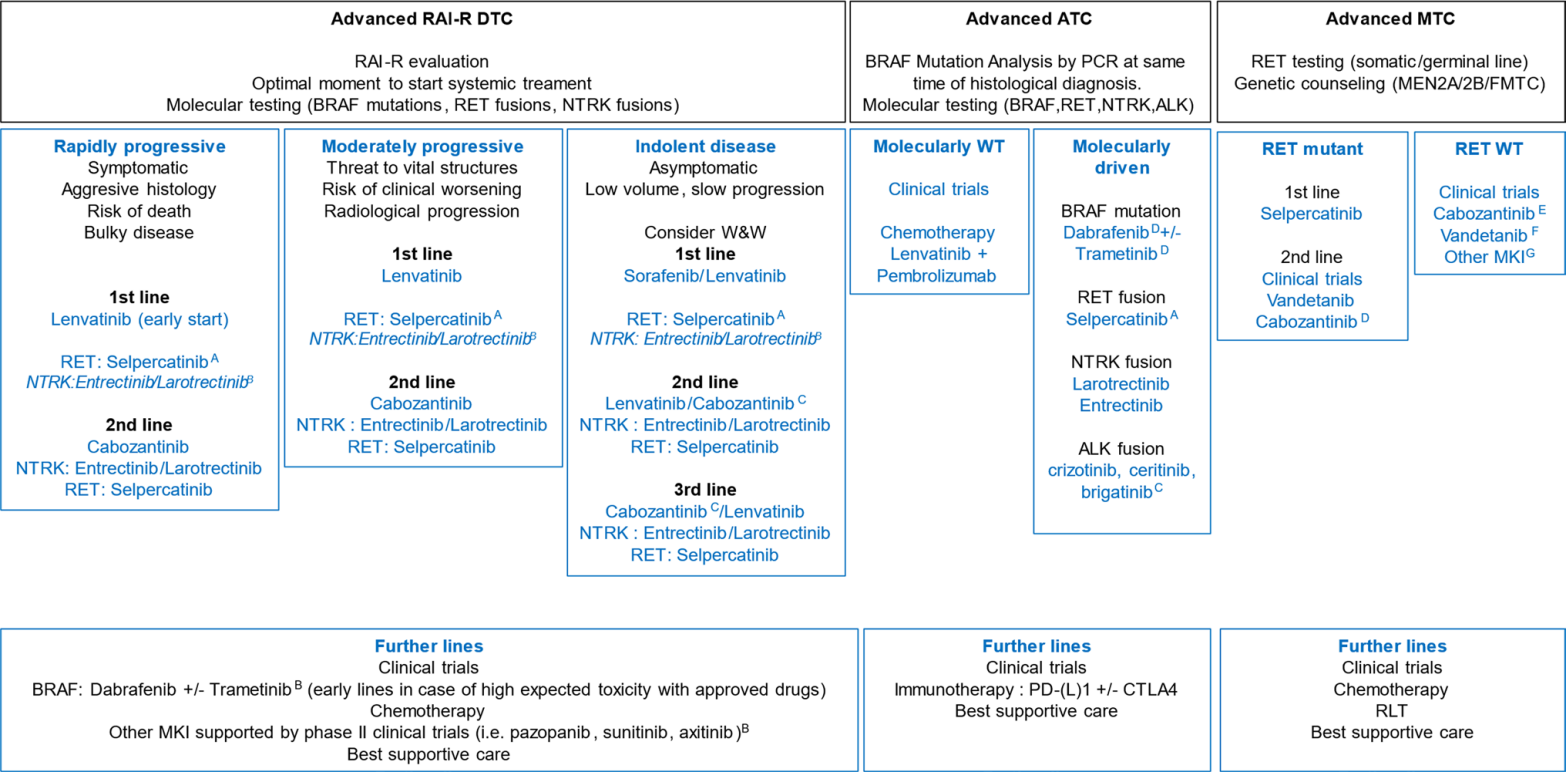
Treatment algorithm DTC, MTC, ATC. *A* Approved in patients after progression to at least one previous line of MKI in Spain, but with EMA approval in first line; *B* Approved in patients without alternate satisfactory options in Spain;* C* Off-label, indication not approved in Spain; D Only FDA approved; *E* EMA approved, not available in Spain; *F* Not recommended by EMA in RET wild type patients. *G* Other MKI, different from vandetanib and cabozantinib, are off-label indication not approved in Spain (i.e., lenvatinib, sunitinib, axitinib, sorafenib or pazopanib), based on small prospective trials, but may represent an option in patients with RET WT MTC. *RAI-R* Radioactive Iodine-Refractory, *DTC* Differentiated Thyroid Carcinoma, *ATC* Anaplastic Thyroid Carcinoma, *MTC* Medullary Thyroid Carcinoma, *MEN2A* Multiple endocrine neoplasia 2A, *MEN2B* Multiple endocrine neoplasia 2B, *FMTC* Familial medullary thyroid carcinoma, *W&W* Watch & Wait, *PCR* Polymerase chain reaction, *WT* Wild type, *MKI* Multikinase inhibitors, *RLT* Radioligand therapy

Systemic treatment is based on multikinase inhibitors (MKI). Three drugs—sorafenib (DECISION trial), lenvatinib (SELECT trial), and cabozantinib (COSMIC-311 trial) have showed positive results in randomized phase III clinical trials with placebo controls [45–47]. Although these studies demonstrated progression-free survival (PFS) benefit, there was no improvement in OS due to crossover effects. Direct comparisons are challenging due to variations in patient population (DECISION for first-line, SELECT for first/second-line and COSMIC-311 for second/third-line) and trial entry criteria. The trials reported different PFS and overall response rate (ORR), comparing MKI with placebo: 10.8 vs 5.8 months and 12.2% vs 0.5% in the DECISION trial, 18.3 vs 3.6 months and 64.8% vs 1.5% in the SELECT trial, 11 vs 1.9 months and 11% vs 0% in the COSMIC-311 trial [45–47] (Fig. 3). Toxicity profile was also different as main grade ≥ 3 (at least in 5% of patients) with sorafenib where hand–foot syndrome (HFS), hypertension, fatigue, weight loss, hypocalcemia; with Lenvatinib where hypertension, diarrhea, fatigue/asthenia, decreased weight and proteinuria and with cabozantinib where HFS, diarrhea, hypertension, fatigue, hypocalcemia. Optimal MKI sequence cannot be determined based on the available evidence, which comes from subgroup analysis from phase III trials. In the SELECT trial, patients receiving first-line lenvatinib achieved a better median PFS of 18.7 months, compared to those receiving lenvatinib as a second line, where the median PFS was 15.1 months (25.6% of patients included) [46]. A subgroup analysis of COSMIC-311 showed that patients experienced better PFS following sorafenib than lenvatinib [48]. Individualized approach should be assessed considering each drug efficacy and toxicity profile, based on the different clinical scenarios (Fig. 3).

Furthermore, we have evidence from different MKI evaluated in phase II studies (i.e., axitinib, sunitinib, pazopanib), and may be considered in situation of refractoriness to previous treatments [49].

#### Molecularly driven options

BRAF mutations, RET fusions, and NTRK fusions are the most common targetable molecular alterations in DTC [13, 14]. Treatment with specific RET and NTRK inhibitors has demonstrated a high ORR. Though not directly compared, data on PFS and ORR showed better results when those selective inhibitors were administered to the treatment-naïve over pretreated cohort of patients [50–52]. Currently, the RET inhibitor selpercatinib is approved following prior treatment with sorafenib and/or lenvatinib, but has recently received approval by EMA in first line based on the LIBRETTO-001 data [53]. The NTRK inhibitors larotrectinib and entrectinib are available for patients without satisfactory treatment alternatives [51, 52].

Only one phase 2 study has explored the treatment of patients with BRAF mutations using dabrafenib as monotherapy vs a dabrafenib–trametinib combination, reaching a high but similar ORR in both arms (42 vs 48%) [54]. While not approved, treatment with dabrafenib or dabrafenib–trametinib may be considered in selected pretreated cases.

Limited evidence exists for the role of selective inhibitors enhancing RAI uptake in RAI-R patients with dabrafenib, vemurafenib, or selumetinib [55–57]. Currently, this approach is considered experimental and restricted to clinical trials.

Recommendations

- Multidisciplinary evaluation should address RAI-R status.
- The optimal timing for initiating systemic therapy and MKI sequence should be individualized for each patient based on the clinical evolution of the disease, including Lenvatinib/Sorafenib in first/second line and cabozantinib in second/third line (Level of evidence: I; Grade of recommendation: A).
- Molecularly driven options include RET fusions (selpercatinib), NTRK fusions (larotrectinib, entrectinib) (Level of evidence III; Grade of recommendation A) and BRAF mutations (dabrafenib, dabrafenib/trametinib) (Level of evidence: II; Grade of recommendation: B).
- Clinical trial enrollment should be promoted to improve patient outcomes.

### Anaplastic thyroid carcinoma

Despite the poor prognosis, molecularly driven options, particularly those targeting BRAF mutations, are crucial to consider. In addition, ALK, NTRK1/3 and RET gene fusion, though infrequent, should be also explored due to several treatment implications [14, 58].

ATC poorly responds to conventional chemotherapy (taxanes, anthracyclines, and platins) with significant toxicities [59]. MKI have limited activity in ATC, including lenvatinib, sorafenib and axitinib [60–62].

According to reports, PD-L1 expression in ATC may range from 22 to 90%. Spartalizumab, an anti-PD-1, demonstrated sustained responsiveness with a 19% ORR in a phase II trial, with better results in patients with higher PD-L1 expression [63]. PD-(L)1 inhibitors in combination with targeted therapies are under research: the phase 2 ATLEP trial with lenvatinib and pembrolizumab in ATC (and PDTC), demonstrated a high ORR, long-lasting remissions, and a tolerable safety profile (NCT02973997) [64]. Other trials are currently ongoing, assessing the combination of atezolizumab with chemotherapy or targeted therapy (NCT03181100).

#### Molecularly driven options

Due to a significant subset of ATC harboring BRAF mutations, BRAF inhibitors (vemurafenib and dabrafenib) have been explored as monotherapy and in combination with MEK inhibitors (trametinib) [65]. On 2018, the FDA approved the combination for BRAF V600E-mutant ATC with an ORR of 59% and significantly improved survival (median PFS 6.7 months, median OS 14.5 months) [66, 67]. Currently, the addition of pembrolizumab to dabrafenib and trametinib has shown promising results that may confer an advantage in survival over the targeted therapy combination alone [68].

In patients with RET-altered ATC, phase I/II clinical trials with selective RET inhibitors (selpercatinib, pralsetinib) have shown long-term effectiveness with acceptable toxicity [53]. Meanwhile, in NTRK-altered ATC, larotrectinib displayed lower activity compared to other TC subtypes (29% ORR, median PFS 2.2 months, median OS 8.8 months) [52]. Despite lack of clinical studies, ALK inhibitors (crizotinib, ceritinib, brigatinib) were reported to be effective for ATC patients harboring ALK fusion [69].

Recommendations

Upfront molecular profiling is strongly recommended to choose the optimal therapy option:

- Dabrafenib–trametinib for BRAF V600E carriers (Level of evidence: II; Grade of recommendation: A).
- Larotrectinib or entrectinib for NTRK fusion (Level of evidence: III; Grade of recommendation: B).
- Selpercatinib for RET fusion (Level of evidence: III; Grade of recommendation: B).

Clinical trial enrollment should be encouraged to improve patient outcomes.

Others:

- Systemic chemotherapy for patients without any target alterations (Level of evidence: IV; Grade of recommendation: B).
- PD-(L)1 inhibitors alone or in combination with targeted therapies, specially lenvatinib–pembrolizumab combination (Level of evidence: III; Grade of recommendation: B) or dabrafenib–trametinib–pembrolizumab (Level of evidence IV; Grade of recommendation: B).

### Medullary thyroid carcinoma

#### Initial treatment

In patients with synchronous metastatic disease at diagnosis of MTC, the role of the primary tumor surgery is under debate. However, it is usually recommended to perform primary tumor surgery (without extended neck dissection) to improve local symptoms and prevent from potential complications [70, 71]. Nevertheless, the high response rate demonstrated by novel directed systemic treatments in advanced MTC, may modify the treatment sequence and surgery indications in this setting [52, 53, 72].

#### Multikinase inhibitors

Vandetanib and cabozantinib are recommended as systemic therapy in patients with progressive and/or symptomatic unresectable locally advanced or metastatic MTC, based on phase III randomized clinical trials. Both showed improved PFS compared to placebo, with ORR between 28 and 45% [73, 74]. According to RET mutational status in the subgroup analysis, the magnitude of benefit in terms of PFS was also different from cabozantinib or vandetanib compared with placebo, showing greater benefit in patients with RET-mutant tumors [73, 74]. Treatment selection should be based on baseline patient characteristics, clinical experience, and different safety profiles. EMA has recently restricted vandetanib use to RET-mutated patients so, the RET non-mutant population has become a great challenge as in some countries, cabozantinib is also not approved.

#### Molecularly driven options

Selpercatinib is indicated for patients with advanced RET-mutant MTC as first-line or progressing to vandetanib or cabozantinib, reaching durable responses with lower side effects compared to these MKI [53, 75]. The LIBRETTO-001 phase I trial showed the efficacy of selpercatinib in patients with MTC who were pretreated and MKI naïve [53]. The phase III trial LIBRETTO-531 trial confirmed a statistically and clinically relevant improvement in PFS and ORR compared to cabozantinib or vandetanib in the first-line setting [75]. Pralsetinib, another selective RET inhibitor, has been approved exclusively by the FDA in 2020 for advanced RET-mutant MTC patients, based on the beneficial outcomes observed in the ARROW study, and no further development in TC is expected [76].

Recommendations

- RET testing is required to assess MEN2 association, which includes genetic counseling, and to choose the best treatment for advanced/metastatic MTC from the first line:
- Selpercatinib for RET-mutant MTC as first-line (Level of evidence: I; Grade of recommendation: A).
- Selpercatinib for RET-mutant MTC after MKI (Level of evidence: II; Grade of recommendation: A).
- Vandetanib (restricted to RET-mutant) or cabozantinib (not approved in Spain) (Level of evidence: I; Grade of recommendation: B).
- Clinical trial enrollment should be promoted to improve patient outcomes.

## Follow-up, long-term implications and survivorship

### Differentiated thyroid carcinoma

Follow-up tools depend on tumor histotype and initial risk of persistent/recurrent disease.

All patients with DTC should have neck US and serum Tg and TgAb assays 6–18 months after primary treatment, and the subsequent follow-up schedule will depend on the estimated risk [19]:

- PTC patients can be monitored with Tg and TgAb and neck US assessments every 12–24 months. For patients showing a biochemical incomplete response, assessments should be conducted every 6–12 months.
- High-risk PTC patients, poorly differentiated thyroid cancer or widely invasive follicular thyroid cancer, serum Tg and TgAb levels should be assessed every 6–12 months.

A whole-body iodine-131 scan is not indicated during follow-up. CT, magnetic resonance imaging (MRI) in some sites or Fluoro-2-deoxy-D-glucose-positron emission tomography (FDG–PET) combined with CT are useful for assessing the extent of disease and is the first-line isotopic imaging technique for patients with RAI-refractory disease [77].

Rising Tg or TgAb levels warrant further imaging studies [78]. For patients at high-risk of recurrence, the absence of circulating Tg may indicate tumor dedifferentiation, and necessitate more intensive follow-up imaging studies [10].

### Medullary thyroid carcinoma

Calcitonin and CEA monitoring are the main tools in the follow-up of patients with MTC. In case of MTC in the context of hereditary syndromes, urine catecholamines are also recommended. Calculation of doubling times, that should be based on at least four consecutive measurements over a 2-year period, will determine the strategy [25].

- In patients with calcitonin and CEA undetectable or within normal range and no structural evidence of disease, serum calcitonin could be measured every 6 months for 1 year, then annually. Neck US should be performed depending on calcitonin levels.
- In patients with detectable and abnormal calcitonin and/or CEA with no structural evidence of disease, no treatment initiation is indicated, but serum calcitonin and CEA should be performed every 3 to 6 months, and depending on those, neck US every 6 to 12 months or other imaging modalities should be considered.

Doubling times shorter than 24 months are associated with progressive disease [79]. Clinically relevant disease sites are rarely detected in patients with calcitonin levels < 150 pg/ml.

Imaging modalities useful to identify locoregional and/or distant metastasis are CT, MRI to identify liver or central nervous system lesions and neck US. MTCs generally display low avidity for FDG, so FDG–PET–CT is not recommended for follow-up, but it can be useful in case of tumor dedifferentiation and rapid progression. F-DOPA–PET has high sensitivity and specificity in MTC and, also 68GaDOTApeptide-PET can be considered before RLT (80).

## Data Availability

Not applicable.
